# Understanding Digital Health Equity: A Conceptual Analysis

**DOI:** 10.1111/jan.70405

**Published:** 2025-11-28

**Authors:** Salsabela Razaq, Saleema Allana

**Affiliations:** ^1^ Western University London Ontario Canada

**Keywords:** concept analysis, digital health equity, digital health technology, nursing, Walker and Avant

## Abstract

**Aim:**

The purpose of this concept analysis is to clarify the meaning of digital health equity beyond a simplistic definition, obtaining a richer meaning that can guide the digital healthcare landscape.

**Background:**

With the growing spread of digital health, digital health equity should be at the center of healthcare. Health outcomes for equity‐deserving groups may be compromised without a clear understanding of digital health equity. Although the concept of ‘health equity’ has been analysed before; no concept analysis has been completed for the concept of ‘digital health equity’.

**Design:**

Concept analysis using Walker and Avant's method.

**Data Sources:**

Articles from PubMed, Scopus and Google Scholar with no limitation on the period of data collection.

**Methods:**

Walker and Avant's concept analysis method was used to outline attributes, antecedents, consequences, and empirical referents of the concept digital health equity.

**Results:**

The main attribute of digital health equity is digital health technology that benefits everyone fairly. The antecedents include: (1) appropriate infrastructure; (2) cognitive abilities including digital literacy; (3) intersectionality of multiple vulnerabilities; (4) presence of the core ethical principles in healthcare; (5) digital accessibility with careful consideration of the social determinants of health; and (6) co‐creation of digital health technologies. The main consequences are improved patient health outcomes and elimination of the digital divide.

**Conclusion:**

This analysis explored the concept of digital health equity as a means to promote positive health outcomes for equity‐deserving groups, highlighting the critical role of nursing practice and research in addressing digital health disparities.

**Impact Statement:**

This paper can have an impact on nursing practice, education and wider social and economic issues. First, various barriers encountered by patients when utilising digital health technologies can be understood. Second, clinicians can be encouraged to assess digital health equity, improve interventions for equity‐deserving groups, and evaluate the effectiveness of digital health interventions to ensure they are equitable. In the context of educational implications, the understanding of digital health equity can be used to facilitate the creation of appropriate education materials for clinicians. Finally, on a wider social and economic scale, understanding digital health equity can aid in the creation of policies to enable equitable digital health technologies.

**Patient or Public Contribution:**

No patient or public contribution because this paper is a concept analysis.

## Summary Statement

**Why is this research or review needed?:**

- Digital health equity is a novel concept with direct links to patient health outcomes.
- A clear conceptual foundation of digital health equity can address existing ambiguities and can inform equitable care within the nursing field.
- Despite the creation of various digital health equity frameworks, the complexities of digital health remains underexplored.

**What are the key findings?:**

- Technology that benefits all individuals fairly is the main attribute of digital health equity.
- The antecedents necessary for digital health equity are as follows: (1) appropriate infrastructure; (2) cognitive abilities including digital literacy; (3) intersectionality of multiple vulnerabilities; (4) presence of the core ethical principles in healthcare; (5) digital accessibility with careful consideration of the social determinants of health; and (6) co‐creation of digital health technologies. Improved patient health and elimination of the digital divide are identified as consequences of digital health equity.

**How should the findings be used to influence policy/practice/research/education?:**

- An in‐depth understanding of digital health equity obtained from this concept analysis can be used to facilitate the creation of appropriate education materials for healthcare providers including nurses.
- The attributes identified in this concept analysis can be used to guide the direction of future research around digital health equity among specific equity‐deserving groups.
- A clear understanding of digital health equity can aid in the creation of policies to enable equitable digital health technologies.

## 1 Introduction

Digital health technologies (DHTs) are advancing at unprecedented rates, and the concept of digital health equity (DHE) is fundamental to ensure all populations, including equity‐deserving groups, can benefit from these technologies (Jaworski et al. 2023; Richardson et al. 2022; Rowland et al. 2024). Without a great emphasis on DHE, inequities such as those caused by limited access and poor digital literacy skills will worsen, ultimately leading to poor patient health outcomes (Lawrence 2022; Lythreatis et al. 2022; Vassilakopoulou and Hustad 2023). It is imperative to first fully grasp the umbrella terms digital health and health equity to understand the concept of DHE (Jaworski et al. 2023; Rowland et al. 2024). Digital health is defined as tools and services that promote health and manage illness through technology (Canadian Association of Schools of Nursing 2022; Ronquillo et al. 2023). Digital health technologies are tools, applications and platforms that leverage technology for healthcare purposes (Yeung et al. 2023). Digital health technology (DHT) broadly refers to the use of mobile health, virtual care, remote monitoring, tools for data exchange, artificial intelligence and more (WHO 2021). Health equity is defined as ‘the fair and just opportunity for all people to achieve their full health potential without variation from personal characteristics, historical oppression and societal influences’ (Lewis et al. 2023, 71). Digital health equity accounts for the diverse needs of all populations with different socioeconomic statuses, ages, races, health conditions, digital literacy levels, and geographic locations (Hadjiat 2023; Jaworski et al. 2023; Lawrence 2022; Vassilakopoulou and Hustad 2023).

### 1.1 International Relevance

Digital health equity is shaped by the digital determinants of health (DDOH) and the social determinants of health (SDOH), which present a significant global challenge, disproportionately affecting equity‐deserving groups (Chidambaram et al. 2024; Crawford and Serhal 2020; WHO 2025a). Equity‐deserving groups, present worldwide, experience health disparities influenced by intersecting factors such as race, gender, disability, immigration, income and education (Burton et al. 2024). Digital health equity is not confined to any specific region; rather, it holds global relevance. A scoping review conducted by Yao et al. (2022) outlined the existing literature documenting the presence of DH inequities across countries around the world. These countries included Australia, Bangladesh, Canada, Indonesia, Israel, Italy, Korea, the Netherlands, Norway, Switzerland, the United Kingdom and the United States (Yao et al. 2022). A study conducted by Yi et al. (2024) revealed DH inequities in South and Southeast Asia, further emphasising the global relevance of DHE.

### 1.2 Situating Digital Health Equity Within the Context of Nursing Knowledge

Health equity is a major concept in nursing practice, research and theory. At an individual nursing level, nurses can alleviate health inequities through effective communication, unbiased care, and supportive strategies that empower patients to manage their own health (Oruche and Zapolski 2020). Achieving health equity also requires nurses to carefully consider the SDOH (American Association of Colleges of Nursing n.d.; Davis 2022). Nursing research plays a critical role in advancing health equity by generating evidence to inform equitable care practices (Still et al. 2023).

Furthermore, health equity is central to nursing theory. Walter's theory of emancipatory nursing praxis (ENP), for example, provides a framework for addressing health inequities, underscoring the theoretical foundations of nursing's commitment to health equity (Velasco and Reed 2023). Walter's theory of ENP is particularly appropriate because it is centred around professional nursing obligations to address barriers beyond individual‐level determinants (Velasco and Reed 2023). Emancipatory nursing praxis (ENP) offers a critical lens through which nurses can engage to promote equitable healthcare (Velasco and Reed 2023). This concept analysis focuses on DHE, an extension of the broader concept of health equity; therefore, an ENP lens can look beyond individual‐level determinants causing inequities, allowing nurses to engage in equitable DH initiatives. As digital health becomes increasingly integral to healthcare delivery, it is essential to examine its relevance to nursing practice, research and theory.

## 2 Background

Digital health equity is a newly emerging concept that has evolved significantly over time, primarily due to its heavy reliance during and post the COVID‐19 pandemic (Crawford and Serhal 2020; Kaihlanen et al. 2022). Despite the increased use of DHTs and the development of various DHE frameworks, the concept of DHE remains unclear (Crawford and Serhal 2020; Jahnel et al. 2022; Lawrence 2022; Richardson et al. 2022). To date, no comprehensive concept analysis has been conducted, which may hinder digital health outcomes for equity‐deserving groups. This underscores the significance of understanding the complexities of DHE. Therefore, the purpose of this paper is to use Walker and Avant's (2011) method of concept analysis to clarify the meaning of DHE beyond a simplistic definition, obtaining a richer meaning that can guide the digital healthcare landscape. Walker and Avant (2011) explain that concepts are mental constructs that are prerequisites for theory development. They outline a systematic framework for analysing a concept when it is undefined or when it has evolved over time. It is important to note that since knowledge is consistently evolving, the findings of a concept analysis are always considered tentative (Walker and Avant 2011). Walker and Avant's (2011) method of concept analysis will be employed to obtain a comprehensive understanding of DHE at this current time. Although there are several approaches to conducting a concept analysis, Walker and Avant's (2011) method will guide this paper due to its clarity, which allows for ease of application. Their method involves eight steps to capture the full essence of the concept, which will be addressed in the analysis section of this paper. These steps include (1) select a concept; (2) determine the aims or purposes of analysis; (3) identify all uses of the concept that can be discovered; (4) determine the defining attributes; (5) identify a model case; (6) identify borderline, related, contrary, invented and illegitimate cases; (7) identify antecedents and consequences; and (8) define empirical referents (Walker and Avant 2011).

## 3 Data Sources

A comprehensive search of the literature was conducted using the electronic databases PubMed, Scopus and Google Scholar. The search included ‘digital health equity’ OR ‘digital health equit*’ OR ‘digital health AND equit*’. To understand how the concept of DHE evolved over time across various disciplines, the date of publication and subject area were left unrestricted. Only articles written in English about DHE were included in this concept analysis. Articles were initially screened for relevance by reviewing the titles and abstracts; if it was deemed suitable to the aims of this concept analysis, the article was read in its entirety. In addition, the reference lists of selected articles were reviewed to identify supplementary relevant research. Moreover, the search included the online Collins (n.d.) and Merriam‐Webster (n.d.) dictionaries. Additionally, the term ‘digital health equity’ was searched via Google to find grey literature from various organisations and government websites. The search was concluded by searching for Dr. Alisson Crawford (who is at the forefront of promoting equity in digital health) in the Google Scholar database.

## 4 Overview of the Concept

### 4.1 Select a Concept

Walker and Avant (2011) outline that the first step in conducting a concept analysis is selecting a concept that is of significance. Conducting a concept analysis on DHE is significant because it builds upon the foundational work done on broader health equity (Lewis et al. 2023). Digital health equity is emerging as a critical and distinct dimension of health equity that warrants its own analysis. Understanding the concept of DHE can support nursing practice, leadership, research, education and policy particularly to ensure that DH advancements do not widen disparities but instead contribute to equitable health outcomes for equity‐deserving groups. Walker and Avant's (2011) method is the most suitable for this concept analysis due to its structured, step‐by‐step approach that facilitates exploration of multifaceted concepts, such as DHE.

### 4.2 Determine the Purposes of Analysis

The second step of a concept analysis proposed by Walker and Avant (2011) is to explicitly state the purpose of the analysis. The purpose of this concept analysis is to clarify the meaning of DHE beyond a simplistic definition, obtaining a richer meaning that can guide the digital healthcare landscape (Hellman 2024).

### 4.3 Identify All Uses of the Concept

Walker and Avant's (2011) third step in the concept analysis involves identifying all uses of the concept. In doing so, this reduces bias and promotes an invaluable understanding of the concept (Walker and Avant 2011). There is no widely accepted definition of DHE (Kim and Backonja 2025). As a result, the terms digital, health and equity were all searched separately.

Digital refers to technology, electronic signals or binary data (Collins n.d.; Merriam‐Webster n.d.). Within the information technology discipline, digital is derived from the Latin term ‘digit’ or ‘finger’ and this is the earliest tool used to count (IGI Global n.d.). When information is either stored, transmitted or forwarded in a digital format it must be converted into a numerical system, known as digital (IGI Global n.d.). The term ‘digital’ often refers to technology that requires the use of microprocessors such as computers or applications that depend on the internet (IGI Global n.d.).

Health is defined as the body's ability to function and overall physical and mental well‐being (Collins n.d.; Merriam‐Webster n.d.). The term health can also be used to describe conditions of a community or system (Collins n.d.; Merriam‐Webster n.d.). The WHO defines health as the state of physical, mental and social well‐being and not just the mere absence of disease (World Health Organization 2025b). The WHO definition of ‘health’ is widely used in medicine and many other disciplines (World Health Organization 2025b). In the context of nursing science and social epidemiology, health is defined as the maintenance or restoration of psychological, cognitive, socioemotional and physical health for the purpose of adopting and managing changes in life (Joannès et al. 2024). Florence Nightingale's definition of health remains in today's nursing practice, defined as ‘not only to be well, but to use well every power we have’ (Beck 2021, 2).

Equity is described as treatment that is fair regardless of individual differences. It also encompasses ownership rights in properties or companies and can appear in law when describing a just system (Collins n.d.; Merriam‐Webster n.d.). Equity can be defined in three ways: as a conceptual construct, benchmark or experience (Plamondon and Shahram 2024). As a conceptual construct, equity is a ‘manifestation of worldview, particularly the ways in which notions of justice and fairness function differentially in society’ (Plamondon and Shahram 2024, 3). As an experience, equity is the feeling of navigating, interacting or socialising with others or with systems and settings (Plamondon and Shahram 2024). Equity as a benchmark means the standard that guides health and social sciences efforts to ensure everyone can live with dignity and their full potential without experiencing systemic disadvantages (Plamondon and Shahram 2024).

A scoping review conducted by Kim and Backonja (2025) presents five explicit definitions of DHE as seen in Table 1.

**TABLE 1:** This table presents multiple definitions of DHE.

Source	Definition
Blanc et al. (2023)	‘the fair and just opportunity to engage with digital health tools to support good health outcomes.’ (262)
Foley et al. (2021)	‘Digital health equity is concerned with fair and just access to, use of and benefit from digital health services and is a critical axis of contemporary health promotion.’ (1105)
Ha et al. (2023)	‘the readiness of all individuals to access digital health, regardless of age, race, income or technology access, ensuring that no one is left behind due to a lack of connectivity or literacy.’ (2)
Kaihlanen et al. (2022)	‘… an equal opportunity for individuals to benefit from the knowledge and practices related to the development and use of digital technologies to improve health.’ (2)
Richardson et al. (2022)	‘… [digital health equity includes] equitable access to digital healthcare, equitable outcomes from and experience with digital healthcare and equity in the design of digital health solutions.’ (2)

The Covid‐19 pandemic accelerated the use of DHTs, which subsequently highlighted the inequities within it, leading to the development of various DHE frameworks (Crawford and Serhal 2020; Hatef et al. 2024; Kim and Backonja 2025; Richardson et al. 2022). The scope of these frameworks is commonly guided by the SDOH and the DDOH to promote equity when using DHTs (Crawford and Serhal 2020; Hatef et al. 2024; Kim and Backonja 2025; Richardson et al. 2022). The DDOH reflect ‘socio‐economic and socio‐cultural context of individuals and the intermediate health factors’ (Kaihlanen et al. 2022, 2).

Furthermore, professional organisations, including the Association of Health Care Journalists (n.d.) and Public Health Ontario (2023), similarly define DHE as the use of DHTs that fosters a fair and equal opportunity for improved health outcomes, while carefully considering the needs of every individual. According to the Centre for Addiction and Mental Health (n.d.‐a) DHE is described as quality healthcare that is delivered through technology for the purpose of improved health benefits without inflicting worse health outcomes for any individual or group.

Beyond the traditional healthcare sector, the American Health Law Association (n.d.) states that DHE is a fair opportunity to attain positive health outcomes when engaging with DHTs. Moreover, the literature refers to DHE as the prevention of unintended consequences from DHTs, such as inequities caused by the digital divide (Crawford and Serhal 2020; Jaworski et al. 2023; Koehle et al. 2022). The digital divide is defined as technology access, usage, and outcome disparities (Lythreatis et al. 2022).

DHE advancements can be evident through numerous indications. For example, when DHE initiatives ensure digital tools use equitable data sources to mitigate risk factors that can exacerbate inequities (Rowland et al. 2024; Yao et al. 2022). Another example is when the design of digital healthcare services incorporates the different needs of individuals (Ha et al. 2023). Additionally, co‐creation with the intended users ensures accessibility and effective utilisation of digital health tools (Koehle et al. 2022). Finally, when DHTs are designed to be user‐friendly and culturally appropriate, it is more likely for equity‐deserving groups to utilise those tools effectively (Wilson et al. 2024).

### 4.4 Determine the Defining Attributes

According to Walker and Avant (2011), the fourth step is to determine the concept's defining attributes. This is achieved by analysing numerous definitions of the concept to extract repetitive characteristics (Walker and Avant 2011). Across all the identified explicit definitions of DHE presented in Table 1, one common attribute is highlighted. The attribute of DHE is DHT that benefits everyone fairly. This attribute is also either explicitly stated or implied in the various DHE frameworks and within the definitions of the related term ‘equity’.

#### 4.4.1 Digital Health Technology That Benefits Everyone Fairly

DHE is a DHT that benefits everyone fairly. This attribute aligns with various definitions of DHE that emphasise fair benefits irrespective of any differences (Public Health Ontario 2023; Lewis et al. 2023; Richardson et al. 2022). These differences include factors, including, but not limited to, SDOH, race, socioeconomic status and health conditions (Hadjiat 2023; Jaworski et al. 2023; Lawrence 2022; Vassilakopoulou and Hustad 2023). There is limited application of DHTs within racialised and Indigenous communities (Public Health Ontario 2023; Saeed and Masters 2021; Yao et al. 2022). For example, an application created to assist physicians in diagnosing skin conditions did not work for those who had Black and Brown skin; therefore, these populations did not benefit fairly from this DHT, when compared to others (Koehle et al. 2022). Furthermore, individuals of low socioeconomic status have been found to report reduced adoption of DHTs due to financial constraints, leading to the unfair distribution of health benefits (Badr et al. 2024; Van de Vijver et al. 2023; Yao et al. 2022). Moreover, individuals with poor health conditions (such as chronic conditions, hearing impairments and arthritis) do not fairly benefit due to their limited physical ability when attempting to use DHTs (Van de Vijver et al. 2023; Yao et al. 2022). Equity is at the forefront of DHTs when there is a fair opportunity for health benefits, regardless of all these differences (Public Health Ontario  2023; Lewis et al. 2023; Richardson et al. 2022).

Furthermore, DDOH are used as guiding principles in DHE frameworks (Crawford and Serhal 2020; Lawrence 2022; Richardson et al. 2022). The DDOH are outlined as

(1) individuals' access to digital resources; (2) use of these resources for health seeking; (3) digital health literacy; (4) beliefs about the potential help or harm of digital health care; (5) values and cultural preferences regarding the use of digital resources; and (6) integration of digital resources into community and health infrastructure. (Kaihlanen et al. 2022, 2)

The DDOH are tightly linked to this attribute as they outline further unique elements that need to be considered for everyone to fairly benefit from DHTs (Lawrence 2022; Richardson et al. 2022).

### 4.5 Model Case

The fifth step of Walker and Avant's (2011) concept analysis is the identification of a model case; a model case is an example of the concept that showcases all of the defining attributes. Construction of a model case is valuable because it is a practical application of the concept's attributes. Consider the following constructed model case: Carlos is a 65‐year‐old male who lives in a rural region of Paris, Ontario. He has unmanaged type 2 diabetes because travelling to his family doctor is financially challenging. Upon his daily morning walk, he noticed a local community health staff member advertising a mobile health application program. This application assists patients in managing their chronic conditions by virtually connecting patients with physicians and providing them with online, tailored resources. When approached, Carlos was hesitant to participate because he was worried about the cost of the application, about using the technology, and his language barrier (he can speak English but prefers to read in his native language, Spanish). The staff member acknowledged these concerns and reassured Carlos that the mobile health application was free and that the program provides a workshop that teaches participants all the basic skills needed to use the application. The staff member also explained that the application offers a multilingual feature, allowing users to choose from various languages, including Spanish. Carlos was impressed with the features and agreed to use this mobile health application. Immediately afterwards, he was provided with the required training, the language settings were switched to Spanish, and he proceeded to use the application with ease. In using this application, Carlos connected with a physician and retrieved tailored resources about diabetes. In doing so, he was able to successfully manage his diabetes.

Digital health technology that benefits everyone fairly, is highlighted in this example. Carlos was able to fairly benefit from this DHT as his diabetes was managed despite his income and language challenges. Carlos did not experience any inequities from this DHT because the workshop taught Carlos how to utilise the technology, addressing his limited digital literacy skills, thus increasing his accessibility.

### 4.6 Identifying Additional Cases

#### 4.6.1 Borderline Case

A borderline case is an example of the concept that has most of the defining attributes but not all of them (Walker and Avant 2011). Consider the following constructed borderline case: Jennifer is a 35‐year‐old woman diagnosed with severe anxiety and a hearing impairment. She works at Bell Canada for minimum wage. Her employer has a partnership with a digital mental health platform that offers free counselling sessions and mental health resources. Jennifer was happy with the platform because it had basic and advanced options, making it easy to navigate (even offered in offline mode when no internet was available). Jennifer also reported some improvement in her anxiety levels from using the platform, but her hearing impairment hindered communication during the counselling service. The technology accounted for various digital literacy levels through basic and advanced user options, and it had an offline mode, which promoted accessibility. However, even though Jennifer had some benefits from using the platform, she was unable to fully benefit from the service as it did not accommodate for her hearing impairment.

#### 4.6.2 Related Case

Related cases are examples of the concept that are related, but do not include all of the defining attributes (Walker and Avant 2011). Consider the following constructed related case: Mariam, a 40‐year‐old physician, used an artificial intelligence (AI) diagnostic tool. This tool was designed to analyse electrocardiograms to identify abnormal cardiovascular rhythms with the aim of promoting better health outcomes for patients. Mariam noticed that they were the only hospital that introduced this tool because it was expensive, and the hospital was well‐funded compared to surrounding hospitals. She also noticed biases in the technology, as the tool predominantly used data from middle‐aged Caucasian men, resulting in reduced accuracy for diagnosing other populations. Mariam also had trouble using this tool because it was very challenging to navigate. This is a related case because although the AI diagnostic tool attempted to improve patient outcomes, it did not benefit all patients fairly. It had biases in the data that disproportionately benefited Caucasian men. This tool created inequities because the hospital did not provide adequate training for Mariam to appropriately use the tool. This case also did not consider various digital literacy levels. Therefore, Mariam was unable to utilise it effectively.

#### 4.6.3 Contrary Case

A contrary case is an example that does not represent the concept (Walker and Avant 2011). Consider the following constructed contrary case: John rushed into a walk‐in clinic during his work lunch break because he could not financially afford to miss a day at work for a scheduled appointment. John experienced longer‐than‐usual wait times because the clinic operated entirely without DHT. Since the wait times were long, John requested a virtual appointment instead; however, the clinic only offered in‐person visits. John left without any support regarding his symptoms because he needed to get back to work on time. Despite leaving in a hurry, he was still late for work, resulting in a pay deduction. This example does not include the defining attributes of DHE. John did not benefit fairly from this service as he left without any guidance for his symptoms due to his financial constraints. John had an inequitable experience because the healthcare was inaccessible.

### 4.7 Antecedents

The seventh step in Walker and Avant's (2011) concept analysis is to identify antecedents and consequences. Antecedents are ‘events or incidents that must occur or be in place prior to the occurrence of the concept’ (Walker and Avant 2011, 167). Any factors or events that must take place to ensure the occurrence of DHE would be considered antecedents of DHE. However, there is a slight difference between the theory and practice of DH. In theory, DH is meant to reduce health inequities; however, in practice, DH may increase inequities due to the absence of antecedents such as appropriate digital literacy skills (Richardson et al. 2022). Therefore, it is important to discuss and understand the antecedents of DHE in depth.

The antecedents necessary for DHE are as follows: (1) appropriate infrastructure; (2) cognitive abilities including digital literacy; (3) intersectionality of multiple vulnerabilities; (4) presence of the core ethical principles in healthcare; (5) digital accessibility with careful consideration of the SDOH; and (6) co‐creation of DHTs. These antecedents have been drawn from various sources used when identifying all uses of the concept. The two major sources are the DHE frameworks and DHE‐related research literature. Guiding frameworks used in the identification of the specific antecedents include the framework for digital health equity developed by Richardson et al. (2022), the digital health equity framework developed by Crawford and Serhal (2020), and the digital healthcare equity framework created by Hatef et al. (2024). Studies undertaken by Ha et al. (2023), Kaihlanen et al. (2022), Kim and Backonja (2025), Koehle et al. (2022) and Wilson et al. (2024) are some of the DHE‐related studies that have been used to identify the antecedents.

As previously discussed, the term ‘digital’ refers to content within a digital format (IGI Global n.d.), and digital health is a form of digitalised healthcare delivery; consequently, infrastructure is a foundational antecedent to this digitalisation (Labrique 2025). There must be appropriate infrastructure available in the community and healthcare settings for DHE to exist. Infrastructure includes, but is not limited to, cellular wireless and broadband access (Richardson et al. 2022). Infrastructure is mandatory for digital health technology to operate, and without the appropriate infrastructure the adoption of DHTs is impossible; hence DHE will not occur (Yeung et al. 2023).

Across various DHE frameworks, such as the commonly cited frameworks by Richardson et al. (2022) and Crawford and Serhal (2020), the presence of one's cognitive abilities greatly influences the use of DHTs. Cognitive abilities are described as basic skills such as listening, communicating, reading and comprehension (Ban et al. 2024). This is a significant antecedent because DHE will be impossible without the appropriate cognitive abilities to utilise the DHTs. Digital literacy is a specific cognitive ability, highlighted in various DHE frameworks and is essential for digital health use (Crawford and Serhal 2020; Richardson et al. 2022). Digital literacy refers to the cognitive and technical skills required to successfully navigate DHTs (Richardson et al. 2022). Without digital literacy, one is unable to make use of DH and thus DHE will not take place. Digital health inequities are frequently documented among individuals of an older age (Badr et al. 2024; Jaworski et al. 2023; Rowland et al. 2024). Older adults (aged over 75) are less inclined to utilise DHTs due to limited digital literacy skills (Van de Vijver et al. 2023; Yao et al. 2022).

Intersectionality of multiple vulnerabilities is another key antecedent for DHE. Digital health equity frameworks, such as that developed by Crawford and Serhal (2020), describe that multiple barriers can be experienced by the same individual. When overlapping vulnerabilities are ignored, the concept of DHE will not take place, reinforcing digital health inequities (Crawford and Serhal 2020). For example, there can be biases in the design of DHTs, making some tools less effective for people of colour, creating a DH inequity (Brewer et al. 2020). This same tool may also further cause biases for those who are female if they do not capture symptoms experienced by women and focus on male symptoms instead. During the development of this DHT, there was limited consideration of the intersectionality of multiple vulnerabilities for a black woman of colour, leading to DH inequities. Meaning intersectionality of multiple vulnerabilities must exist before DHE can occur.

The core ethical principles in healthcare are beneficence, nonmaleficence, autonomy and justice (Varkey 2021). These four core ethical principles are not directly linked to the definitions of DHE within the literature, but are implied within DHE frameworks (Crawford and Serhal 2020; Richardson et al. 2022). These four principles are ethical matters that contribute to and maintain health equity and the elimination of the digital divide gap and are thus foundational to the occurrence of DHE. For example, beneficence is promoting benefit to the patient (Varkey 2021). When the principle of beneficence is applied within the healthcare field, equity is possible. Nonmaleficence is the avoidance of harm (Varkey 2021). Nonmaleficence is the obligation of a healthcare provider not to harm the patient (Varkey 2021). When there is active consideration to prevent harm, equity is possible (Varkey 2021). Autonomy entails acts that allow for a patient's self‐determination (Varkey 2021). Autonomy relates to DHE as the patient should make the choices surrounding their digital healthcare while understanding all risks and benefits. Justice is the treatment of a person that is fair, equitable, and appropriate (Varkey 2021). The fair, equitable, and appropriate treatment of a patient is a prerequisite to DHE.

Digital accessibility is commonly cited as a requirement for DHE and is found within explicit definitions of the term by Foley et al. (2021), Ha et al. (2023) and Richardson et al. (2022). Accessibility refers to the ability of an individual and/or population to effectively obtain access to DHTs (Lawrence 2022; de Lopez Coca et al. 2022; Lythreatis et al. 2022). Accessibility is a foundational requirement for the use of DH and therefore, must be in place prior to the occurrence of DHE. DHE is commonly described by the Association of Health Care Journalists (n.d.), Centre for Addiction and Mental Health (n.d.‐b), Ha et al. (2023) and Public Health Ontario (2023) as leaving no one behind, irrespective of any SDOH. The SDOH include employment, income, working conditions, housing, education, social inclusion, and the environment (Canadian Nurses Association 2025). Digital health equity requires more than just access; it demands the careful consideration of the SDOH. For instance, stable housing, a SDOH, is a foundational component for DH access. DH inequities are frequently documented among individuals residing in rural communities (Badr et al. 2024; Jaworski et al. 2023; Rowland et al. 2024). Rural communities can experience an unequal distribution of DHTs due to limited availability and accessibility (Badr et al. 2024; Jaworski et al. 2023; Rowland et al. 2024; Yao et al. 2022). On the other hand, individuals living in rural communities with limited access to healthcare services can receive healthcare via DHTs, which is a pivotal factor for the concept of DHE to occur (Jaworski et al. 2023; Lawrence 2022). Income, another SDOH, directly influences an individual's ability to afford internet or devices to access DHTs. When individuals are unable to afford this technology due to financial constraints, they are excluded from DH, which leads to disparities in access to DHTs. Therefore, careful consideration of the SDOH is essential to ensuring accessibility and achieving DHE (Crawford and Serhal 2020).

There are also antecedents of DHE that take place at the healthcare provider level or healthcare organisational level. For example, how DHT is developed, how the DHT is disseminated and how much support healthcare providers receive in using these DHTs with equity‐deserving groups (Ha et al. 2023; Koehle et al. 2022; Wilson et al. 2024). Each of these examples impacts whether DHE occurs or not. If DHTs are co‐created with underserved communities, then there is a higher likelihood that the technology will be more acceptable to the specific equity‐deserving groups (Blasimme et al. 2025). Similarly, if the DHT is widely disseminated with equity‐deserving groups, there is a greater chance of achieving DHE for everyone (Baumann et al. 2023). If optimum support to healthcare providers is provided, guiding the equitable utilisation of DHTs among different populations, this can ensure the occurrence of DHE widely (Borges do Nascimento et al. 2023).

All antecedents must be present individually for the occurrence of DHE to take place. However, given the nature of the antecedents, they also intersect and can occur in combination for the occurrence of the concept. For example, income (a SDOH) is a requirement to afford DH devices; however, income is also closely tied to accessibility. If one does not have the income to afford digital devices, they may not be able to access DHTs. Another example is as follows: if an individual cannot afford to pay for a smartphone or device to run the technology or for the internet, then it will not be possible to use it despite the presence of the appropriate infrastructure, and all this comes before the concept of DHE. These are just some of the many examples of intersecting antecedents.

### 4.8 Consequences

Walker and Avant (2011) define consequences as the result of a concept. The literature reveals that the main consequences of DHE are improved patient health outcomes and elimination of the digital divide (Crawford and Serhal 2020; The Lancet 2021; Lawrence 2022). The definitions of DHE commonly describe the result of DHE as the attainment of positive health outcomes, as seen in Table 1. For example, DHE promotes a personalised approach to care (Butcher and Hussain 2022), which leads to better patient self‐management of diseases (Abraham et al. 2023) to potentially improve patient health outcomes. Further supporting examples include better disease management, improved diagnosis accuracy, and fewer medical complications leading to reduced morbidity and mortality (Alotaibi and Federico 2017; Awad et al. 2021). Another consequence of DHE is when DHT do not cause or amplify the digital divide (Koehle et al. 2022; Rowland et al. 2024). The digital divide is often linked with DH inequities within the body of literature surrounding DHE (Koehle et al. 2022; Public Health Ontario 2023; WHO 2021). When DHTs account for the two central elements of the digital divide, accessibility and digital literacy levels, to avoid causing inequities or contributing to the existing inequities, this is a consequence of DHE (Jaworski et al. 2023; Lawrence 2022; Vassilakopoulou and Hustad 2023; WHO 2021). The presence of the antecedents brings forth the consequences. For example, the presence of the appropriate infrastructure, digital literacy skills, and accessibility are part of the elimination of the digital divide. The consideration of SDOH and core ethical principles in healthcare helps improve health outcomes for all.

### 4.9 Define Empirical Referents

The last step in Walker and Avant's (2011) concept analysis involves establishing empirical referents. Walker and Avant (2011) emphasise that empirical referents are not tools that measure the concept but rather a way to recognise the presence of the concept's defining attributes. The attribute of DHE is DHT that benefits everyone fairly. An empirical referent for this attribute is when DHTs are designed to meet the needs of diverse patient populations, acknowledging individual differences (Hadjiat 2023; Jaworski et al. 2023; Lawrence 2022; Vassilakopoulou and Hustad 2023). Acknowledging differences can be evident in many ways. For example, if mobile health applications provide tailored health resources based on personal intake questionnaires to effectively accommodate individual patient differences (Badr et al. 2024). These accommodations can include how many interfaces are available within a DHT (basic and advanced options) or different delivery formats such as audio or reading options (Agency for Healthcare Research and Quality 2024). Equity‐deserving groups come from many different cultural and socio‐economic backgrounds and have different preferences and needs, which is why different modes are helpful for underserved communities. Additionally, if the DHT is available in multiple languages (Agency for Healthcare Research and Quality 2024), this is an empirical referent. Different languages within DHTs are needed because not everyone is proficient in the common language of the country they reside in.

## 5 Discussion and Recommendations

It is evident that grasping the concept of DHE in its entirety is essential in the digital healthcare space, particularly in nursing (Badr et al. 2024; Jaworski et al. 2023; Lawrence 2022; Richardson et al. 2022; Rowland et al. 2024). Following this concept analysis, recommendations for practice, education, research, and policy can be identified.

Nursing practice should involve assessing, promoting, and evaluating DHE among equity‐deserving groups; therefore it is necessary to create the infrastructure and framework to make these assessments. As frontline providers, nurses are positioned to influence health outcomes and address digital disparities directly (National Academies of Sciences, Engineering and Medicine 2021). Factors contributing to the digital divide should be assessed by all nurses, as these can cause grave consequences of digital despair (Crawford and Serhal 2020; The Lancet 2021; Lawrence 2022). DHTs must be co‐created with equity‐deserving groups so that their contextual cultural, and socio‐economic realities could inform the co‐creation of these tools and could make these more accessible for all (Baumann et al. 2023; Blasimme et al. 2025; Borges do Nascimento et al. 2023). DHTs must be disseminated widely with equity‐deserving groups by healthcare professionals especially by nurses who work directly with these patients at the front line (Baumann et al. 2023; Blasimme et al. 2025; Borges do Nascimento et al. 2023). Healthcare professionals, including nurses, must be provided with adequate support by their organisations to be proficient in the use of DHTs themselves and to be able to use these DHTs with everyone effectively (Baumann et al. 2023; Blasimme et al. 2025; Borges do Nascimento et al. 2023).

This concept analysis identified various antecedents required for the occurrence of DHE. These findings should urge nurse educators to prepare nurses to assess all the antecedents when they are caring for patients. The WHO (2021) proposed an action plan that advocates for the implementation of equitable DHTs in the healthcare system. In the context of educational implications, the in‐depth understanding of DHE obtained from this concept analysis can be used to facilitate the creation of appropriate education materials incorporated in this action plan and within nursing education. These educational materials can be in the form of training sessions, modules, pamphlets and so on. They can be provided to any healthcare provider that uses or develops DHTs, and/or for healthcare providers who coach patients on how to use DHTs (WHO 2021).

This concept analysis can be used to guide the direction of future research. Since DHE is an emerging concept, its attributes require more attention in research (Jaworski et al. 2023; Koehle et al. 2022; Ronquillo et al. 2023). For example, studies can be undertaken to examine effective ways to promote each attribute within this concept analysis. Future nursing studies should aim to advance DHE by focusing on the antecedents identified in this analysis. Future DHE research should also include the voices of marginalised and underserved communities. The theory of ENP offers a valuable framework to guide such research, as demonstrated by a grounded theory study that explored nursing's role in promoting social justice through the lens of ENP (Rooddehghan et al. 2019). Thus, the theory of ENP can be leveraged in future DHE‐related research within the nursing discipline. Additionally, future research should focus on the co‐creation of DHTs with equity‐deserving groups (Blasimme et al. 2025).

Moreover, the clear understanding of DHE provided in this concept analysis can aid in the creation of policies to enable equitable DHTs. For example, policies can be developed to ensure DHE is achieved by enforcing the use of DHE frameworks when creating DHTs (Crawford and Serhal 2020; Kaihlanen et al. 2022; Lawrence 2022; Richardson et al. 2022). It is important to note that the concept of DHE, along with its antecedents and consequences, reflect a middle range explanatory theory, because there is a clear relationship between the outlined antecedents and consequences, and the concept of DHE. Since the consequences are linked to the antecedents and the antecedents can all intersect, policies should be developed to address the complexity of DHE.

### 5.1 Novelty of This Concept Analysis

This concept analysis expanded the understanding of the concept DHE by not only addressing SDOH, access and literacy, which are frequently discussed, but also bringing in the unique pieces of intersectionality. This concept analysis identifies that each antecedent can impact the consequences of DHE; however, all the antecedents can also intersect with one another. This concept analysis links intersectionality to DHE, which has not been brought to light previously. Additionally, the linkage of the four healthcare ethical principles is not directly cited in DHE literature; however, these four principles are core to DHE and healthcare in general. There needs to be an explicit connection between these principles and DHE.

## 6 Limitations

A limitation of this scoping review stems from the absence of a universally accepted definition of DHE (Kim and Backonja 2025). Consequently, the existing literature employs various DHE frameworks, leading to inconsistencies in the understanding of DHE. The existing body of knowledge is not unified, which prevents a straightforward understanding of the attributes of DHE extending into practical limitations that can lead to digital healthcare inconsistencies. Due to resource, time, and other constraints it is not always possible to access and translate non‐English evidence. Using only English literature in this concept analysis assumes that only evidence written in English is worthy. However, in doing so, this may contribute to further inequities. Moreover, only those who know English or can afford translations will publish in English regarding this topic. This concept analysis is also limited by its reliance on existing literature, which may underrepresent equity‐deserving voices. Furthermore, given the rapidly evolving nature of digital health, emerging definitions and frameworks on DHE may not yet be captured in the literature, potentially limiting the completeness of the concept attributes identified. Therefore, the authors plan to update this concept analysis as the literature in this area evolves further.

## 7 Conclusion

In conclusion, a rigorous concept analysis was conducted to clarify the meaning of DHE. Digital health equity is a multifaceted concept. The findings highlighted that DHE is a concept that benefits everyone fairly. Digital health equity attributes were highlighted in various constructed cases. Various antecedents were identified that work independently and intersect with one another. Improved patient health outcomes and elimination of the digital divide are the consequences of DHE. Digital health equity has implications in practice, education, research and policy. Practice implications involve co‐creation and dissemination of DHTs with equity‐deserving groups and provision of adequate support for healthcare professionals including nurses for the optimum utilisation of these DHTs with equity‐deserving groups. Education implications entail preparing nurses to assess the antecedents of DHE. Future nursing research should aim to advance DHE by focusing on the antecedents of DHE, and research should be inclusive of equity‐deserving groups through co‐creation. Finally, DHE policies can be developed using a combination of various DHE frameworks and based on the relevant research findings.

## Funding

The authors have nothing to report.

## Conflicts of Interest

The authors declare no conflicts of interest.

## Data Availability

Data sharing not applicable to this article as no datasets were generated or analysed during the current study.
